# Gender dimensions of health-related challenges among urban poor during COVID-19 pandemic in low-and middle-income countries: a systematic review and gap analysis

**DOI:** 10.3389/fpubh.2023.1170386

**Published:** 2023-06-09

**Authors:** Krushna Chandra Sahoo, Sapna Negi, Pranab Mahapatra, Kajal Samantaray, Girish Chandra Dash, Shubhankar Dubey, Mili Roopchand Sahay, Rakesh Kumar Sahoo, Debdutta Bhattacharya, Banamber Sahoo, Subhada Prasad Pani, Mariam Otmani del Barrio, Sanghamitra Pati

**Affiliations:** ^1^Health Technology Assessment in India, Indian Council of Medical Research (ICMR)-Regional Medical Research Centre, Bhubaneswar, Odisha, India; ^2^Department of Psychiatry, Kalinga Institute of Medical Sciences, Bhubaneswar, Odisha, India; ^3^Independent Consultant, Bhubaneswar, India; ^4^UNICEF/UNDP/World Bank/WHO Special Programme for Research and Training in Tropical Diseases (TDR), World Health Organization, Geneva, Switzerland

**Keywords:** urban poor, gender, sex, inequities, pandemic, LMICs

## Abstract

The COVID-19 pandemic has varying effects on men, women, and the transgender population. However, there is a paucity of systematic evidence on how gender and other social determinants of health during COVID-19 are affected in resource constraint urban settings. This review describes the gender dimensions of health-related challenges among the urban poor during COVID-19 in LMICs. We searched 11 scholarly online repositories including PubMed, Embase, Web of Science, CINAHL using the domain “slums,” “COVID-19”, “LMICs” and “gender identities.” We used thematic framework analysis to synthesize qualitative data, and meta-analysis to determine the pooled prevalence. We registered in PROSPERO (CRD42020203783). We identified 6490 records, and 37 articles included. The studies reported stress among 74% women and 78% men, depression among 59% women and 62% men, and anxiety among 79% women and 63% men. Men had more stress than women during COVID-19; men are primarily responsible for household sustenance. Women had more anxiety than men, possibly because they are often the primary caregivers for children and the older population. While the severity varies according to gender identity, their vulnerability mostly related to their literacy and economy, highlighting the significance of including all social determinants in future primary studies.

**Systematic review registration:**

https://www.crd.york.ac.uk/prospero/#recordDetails.

## 1. Introduction

Over half of the world's population is currently living in urban areas, which is anticipated to rise to 68% by 2050. The global urban population has expanded at a breakneck pace, from 751 million in 1950 to 4.2 billion in 2018 (1). According to the United Nations Department of Economic and Social Affairs, low-and middle-income countries (LMICs) would account for more than 90% of anticipated urban population growth (1, 2). The urban poor in LMICs, who comprise a large portion of the urban population in these settings, often live in slums or on the streets (3). They have substandard housing conditions shared by many people, as well as a dearth of basic amenities. Additionally, overcrowding, and poor living conditions contribute to an increased risk of developing infectious diseases (4–6). Furthermore, poverty, gender inequality, and health inequities significantly contribute to the burden of infectious diseases (7, 8). Thus, urban health development programmes encounter numerous obstacles in their initiatives to boost the health and wellbeing of the urban poor (3, 9), highlighting the critical need for a comprehensive understanding of their healthcare services during the COVID-19 pandemic (10).

Generally, poor health outcomes among the urban poor were most frequently connected with living conditions, low income, food insecurity, and a lack of social support (4). Additionally, the COVID-19 pandemic increased their vulnerability (11). The COVID-19 poses potential challenges to livelihoods and health care demands, particularly LMICs (12, 13). Consequently, wage loss, societal and gender inequity exacerbated gender-based violence (14). Additionally, women's health challenges including interruptions in reproductive health care have been documented to increase during pandemics (15, 16). It may be difficult to receive affordable, high-quality health care in locations where there is no universal health coverage and primary health care access is already limited and digital health care is not prioritized (12, 13). The adverse health consequences in slums during COVID-19 may be worsened by limited access to health care; highlighting the critical significance of systematic evidence on pandemic associated vulnerability among the urban poor.

Among various social determinants, socioeconomic status was the most frequently reported (17), while gender identity and associated gender dimensions were the second most frequently associated with poor health and wellbeing (18). In most of the LMICs, generally, women were more susceptible than men (19); women living in slum regions are more likely to require health care (18, 19). The COVID-19 pandemic is aggravating gender and sex-related differences in health. Few reports have concluded that the COVID-19 pandemic had a disproportionately impact on women and their employment chances compared to men. However, systematic evidence on how gender and other social determinants affect the health of urban poor during the COVID-19 pandemic in LMICs is still insufficient. Therefore, this review described the sex and gender dimensions of health-related difficulties among the urban poor and their management approaches during COVID-19.

## 2. Methods

### 2.1. Search strategy and selection criteria

We reported this systematic review following the Preferred Reporting Items for Systematic Reviews and Meta-Analyses (PRISMA 2020) guideline (20). PROSPERO has registered for this study (CRD42020203783). Four reviewers (KCS, SD, SN, MS) conducted a thorough search of 11 scholarly online repositories for relevant articles published between November 2019 and August 31, 2021 – PubMed/MEDLINE, Embase, Web of Science, CINAHL (EBSCO), ProQuest, Cochrane, Epistemonikos, WHO Global Index Medicus, MedRxiv and BioRxiv, 3ie Impact Evaluation Repository, and Google scholar. Initially, we created a broad search string that included the terms slums, COVID-19, LMICs and gender identity. We have included all of the countries on the World Bank list classified as LMICs. The strategies for a detailed search are provided (Appendix I).

All curated studies were then imported into EndNote X8 software to identify and remove duplicate records. We imported all EndNote records into Rayyan, a free web tool for the title and abstract screening. Three reviewers independently screened all articles (KCS, SN, and KS). Commentaries, perspectives, reviews, and editorials were not included. Two reviewers (SN and KS) independently review the full text to ensure compliance with the study's objectives. Two reviewers resolved disputes over study inclusion at each stage (KCS and PM). We excluded articles that did not include sex-segregated data during the full-text review.

### 2.2. Data extraction, quality assessment and synthesis

We extracted quantitative data in Microsoft Excel using a standardized template. The data included the study type, the country and city of studies, the types of urban poor, the sex and gender identity of the study population—man, woman and transgender population, the data collection method, and the major domains. Two reviewers (SN and KS) separately extracted data, then cross-checked and compiled by a third reviewer (KCS).

We used the thematic framework analysis approach to synthesize qualitative findings (20, 21). Three authors (KCS, SN, KS) thoroughly reviewed the selected studies, and finally, the author (KCS) developed a framework for data coding. The authors (SN) coded the data and extracted the key findings using MAXQDA software (MAXQDA Analytics Pro 2020, VERBI GmbH Berlin, Germany). We developed a conceptual framework to present the results based on the key findings. We used meta-analysis using random-effects models with MetaXL software Version 5.3 to determine the pooled prevalence of stress, anxiety and depression among women and men use in a forest plot.

Two reviewers (SN and KS) assessed the quality of the studies included. A disagreement concerning the appraisal quality was settled by a discussion with a third reviewer (KCS). We employed the mixed-method-appraisal tool (MMAT) to assess their quality (22) (Appendix II).

## 3. Results

**Figure 1:**
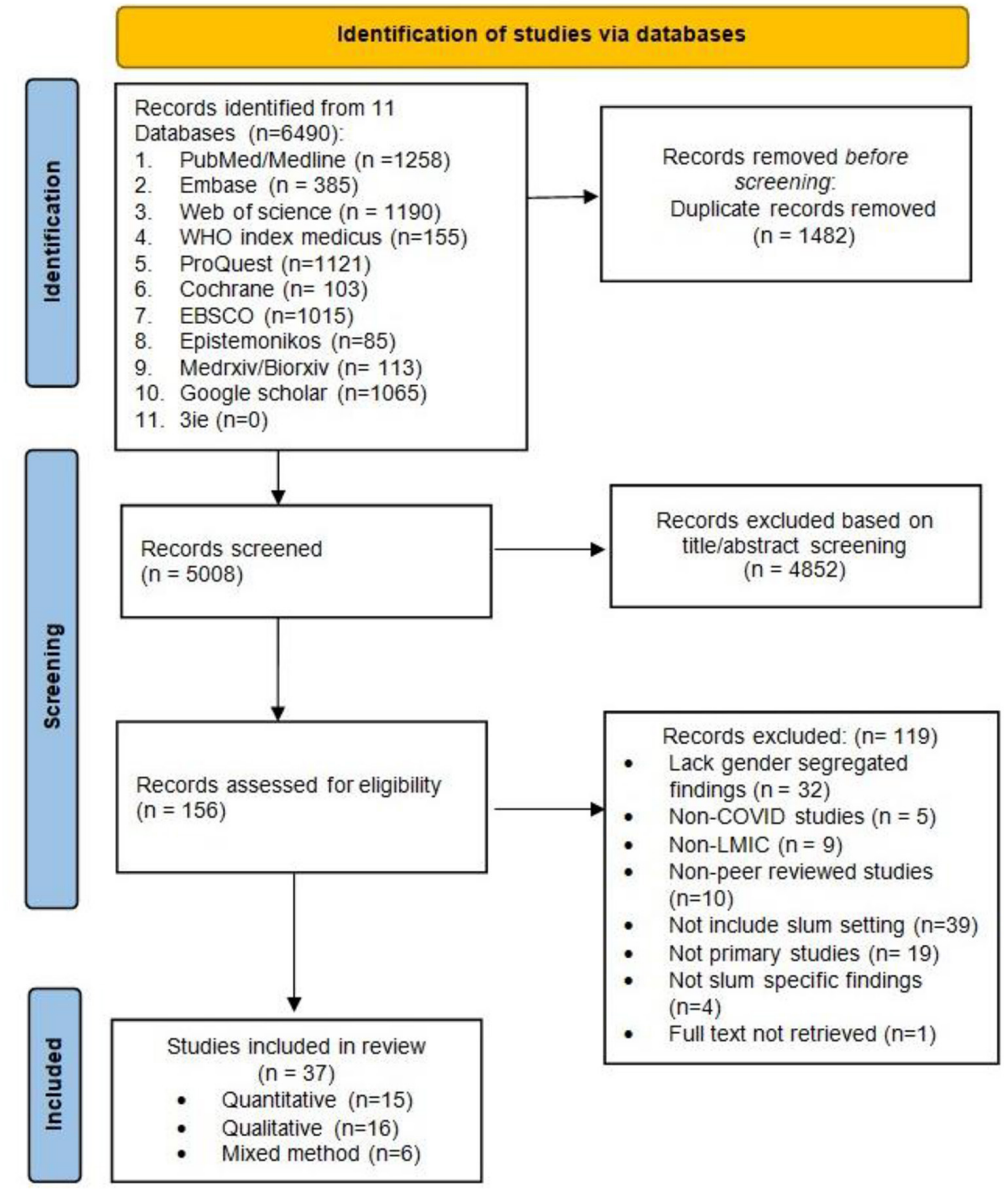
PRISMA flowchart.

We identified 6,490 records, removed 1,482 duplicate records, and selected 5,008 articles for screening. After reviewing 156 full-text articles, finally, we included 37 articles. The PRISMA flowchart illustrates the article selection process (Figure 1). There was no quantitative research relating to gender identities other than the binary categories of men and women. Three qualitative studies among transgender population and hijras were conducted: two in India (23, 24) and one in Bangladesh (25). Table 1 contains detailed features of qualitative studies, while Table 2 contains characteristics of quantitative studies, comprising disaggregated data by sex and gender identity.

**Table 1:** Characteristics of qualitative studies comprising disaggregated data by sex and gender identity.

**Authors year**	**Study type**	**Country**	**City**	**Types of urban poor**	**Study participants**	**Study approach**	**Data collection methods**	**Data analysis**	**Major domain**
Akter et al. (26)	Qualitative	Bangladesh	Coax Bazar	Refugees	Female camp dwellers and leaders (*n =* 66)	Phenomenology	Telephonic Interview	Thematic	Resilience
Arora and Majumder (27)	Qualitative	India	Delhi	Migrant workers	Women (*n =* 5)	Narrative	Telephonic Interview	Narrative	Gender inequality
Azeez et al. (28)	Qualitative	India	Delhi and Gurugram	Migrant workers	Women (*n =* 19)	Phenomenology	Semi-structured Interview	Thematic	Economic crisis and livelihood
Banerjee and Rao (23)	Qualitative	India	Various cities of Karnataka	Transgenders (hijras)	Transgender (*n =* 10)	Narrative	Telephonic Interview	Thematic	Social and emotional risk
Douedari et al. (29)	Qualitative	Syria	Northwest Syria	Displaced Syrian	Refugees (*n =* 20, 7 male and 13 female)	Phenomenology	Telephonic Interview	Thematic	Prevention response
Gichuna et al. (30)	Qualitative	Kenya	Nirobi	Female sex workers	Female sex worker (*n =* 117)	Phenomenology	In-depth Interview	Thematic	Gender-based violence
Jalil et al. (25)	Qualitative	Bangladesh	Dhaka	Transgenders (hijras)	Transgender (*n =* 22)	Phenomenology	In-depth telephone interviews	Thematic	Psychosocial health and discrimination
Kar et al. (31)	Quantitative	India	Bhubaneswar	Slum dwellers	Women (*n =* 280)	Phenomenology	Semi-structured Interview	Thematic	Gender-based violence
Lusambili et al. (32)	Qualitative	Kenya	Nairobi	Refugees	Pregnant woman (*n =* 10)	Narrative	In-depth Interview	Thematic	Healthcare access and economic challenges
Mathias et al. (33)	Qualitative	India	Dehradun	Slum dwellers	Community members (*n =* 24)	Phenomenology	In-depth Interview	Framework	Psychosocial health and Economic crisis
Munajed and Ekren (34)	Qualitative	Syria	From Turkey and Lebanon	Syrian refugees	Refugee family (*n =* 11), NGOs Staff (*n =* 72)	Phenomenology	Semi-structured Interview	Thematic	Housing and health
Nanda (35)	Qualitative	India	Cuttack	Domestic workers	Women (*n =* 100)	Phenomenology	interviews	Thematic	Economic crisis and health
Oluoch-Aridi et al. (36)	Qualitative	Kenya	Nairobi	Slum dwellers	Women (*n =* 72)	Phenomenology	In-depth Interview	Thematic	Reproductive health
Pandya and Redcay (24)	Qualitative	India	Ahmedabad	Urban poor	Transgender Women and Hijra (*n =* 12)	Narrative	Telephone interviews	Thematic	Health care access
Rashid et al. (37)	Qualitative	Bangladesh	Khulna	Slum dwellers	Community members (*n =* 51, 44 Women and 7 men)	Phenomenology	Telephone Interviews	Framework	Livelihood and health
Zakar et al. (38)	Qualitative	Pakistan	Punjab	Slum dwellers	General public (*n =* 21), and Healthcare professionals (*n =* 13)	Phenomenology	Telephone Interviews	Thematic	Economic crisis and health
Akter et al. (26)	Mixed method	Bangladesh	Khulna	Slum dwellers	Community members (*n =* 32) and Service providers (*n =* 10)	Explanatory	In-depth Interview	Framework	Psychosocial health and Economic crisis
Dyalchand et al. (39)	Mixed method	India	Pune	Slum dwellers	Community volunteers, Mahila Arogya Samiti members and frontline workers (*n =* 20)	Explorative	In-depth Interview	Thematic	Psychosocial health and Economic crisis
Guglielmi et al. (40)	Mixed method	Bangladesh	Coax Bazar	Refugees	Male and female adolescents (*n =* 34)	Convergent parallel	Telephone Interviews	Thematic	Livelihood and health
Karp et al. (41)	Mixed method	Kenya	Nairobi, Kilifi and Kisumu	slum dwellers	Adolescent girls (*n =* 57)	Convergent parallel	Telephone Interviews	Thematic	Economic crisis and health
Napier-Raman et al. (42)	Mixed method	India	Delhi	Slum dwellers	Young women (*n =* 9)	Convergent parallel	In-depth Interview	Thematic	Economic crisis and health
Sumalatha et al. (43)	Mixed method	India	Delhi, Mumbai and Kochi	Domestic workers	Women (*n =* 12)	Convergent parallel	In-depth Interview	Thematic	Economic crisis and health

**Table 2:** Characteristics of quantitative studies and prevalence of mental health outcomes comprising disaggregated data by sex and gender identity.

**Authors, year**	**Study type**	**Country**	**City**	**Types of urban poor**	**Population (N)**	**Urban poor (n)**	**Men (n)**	**Women (n)**	**Mental health outcomes**					
									**Stress**		**Anxiety**		**Depression**	
									**Women** ***n*** **(%)**	**Men** ***n*** **(%)**	**Women** ***n*** **(%)**	**Men** ***n*** **(%)**	**Women** ***n*** **(%)**	**Men** ***n*** **(%)**
Afridi et al. (44)	Quantitative	India	Delhi	Informal sector	1,473	1473	740	723	643 (89)	629 (85)	593 (82)	474 (64)	506 (70)	488 (66)
Aguilar Ticona et al. (45)	Quantitative	Brazil	Salvador	Slum dwellers	985	985	394	591	NR	NR	NR	NR	NR	NR
Alonzo et al. (46)	Quantitative	Guatemala		Urban poor	295	295	105	190	74 (39)	43 (41)	89 (47)	67 (64)	22 (12)	24 (23)
Cobre et al. (47)	Quantitative	Brazil	Rio de Janeiro	Urban poor	3,656	2,738	1,257	1,454	NR	NR	NR	NR	NR	NR
Das et al. (48)	Quantitative	India	West Bengal	Returnee migrant worker	159	40	0	40	NR	NA	NR	NA	NR	NA
Islam et al. (49)	Quantitative	Bangladesh	Dhaka	Slum dwellers	435	435	238	197	NR	NR	NR	NR	NR	NR
Jayatissa et al. (50)	Quantitative	Sri Lanka	Colombo	Underserved settlements	236	236	0	127	NR	NR	NR	NR	NR	NR
Kumar et al. (51)	Quantitative	India	Chandigarh	Migrant workers	98	98	98	0	NA	84 (86)	NA	50 (51)	NA	72 (74)
Mamun and Fatima (52)	Quantitative	Bangladesh	Dhaka	Slum dwellers	434	434	289	145	NR	NR	NR	NR	NR	NR
Muhula et al. (53)	Quantitative	Kenya	Kibera	Informal settlements (PLHIVs)	176	176	62	114	NR	NR	NR	NR	NR	NR
Mukhopadhyay (54)	Quantitative	India	Kolkata	Slum dwellers	282	282	144	138	NR	NR	NR	NR	NR	NR
Pinchoff et al. (55)	Quantitative	Kenya	Nairobi	Informal settlements	2,009	2,009	747	1,262	NR	NR	NR	NR	NR	NR
Quaife et al. (56)	Quantitative	Kenya	Nairobi	Informal settlements	213	213	106	108	NR	NR	NR	NR	NR	NR
Santana et al. (57)	Quantitative	Brazil	São Paulo	Urban poor	495	495	47	448	303 (68)	32 (68)	339 (76)	36 (76)	289 (65)	30 (65)
Spiritus-Beerden et al. (58)	Quantitative	Global online survey		Refugees and migrants	20,642	20,642	1,1946	8,696	NR	NR	NR	NR	NR	NR
Dyalchand et al. (39)	Mixed method	India	Pune	Slum dwellers	165	165	0	165	78 (47)	NA	139 (84)	NA	96 (58)	NA
Karp et al. (41)	Mixed method	Kenya	Nairobi, Kilifi, Kisumu	Slum dwellers	756	756	0	756	NR	NR	NR	NR	NR	NR
Napier-Raman et al. (42)	Mixed method	India	Delhi	Slum dwellers	122	122	52	70	51 (73)	14 (27)	NR	NR	NR	NR
Sumalatha et al. (43)	Mixed method	India	Delhi, Mumbai, Kochi	Domestic workers	260	260	0	260	NR	NA	234 (90)	NA	NR	NA

**Figure 2:**
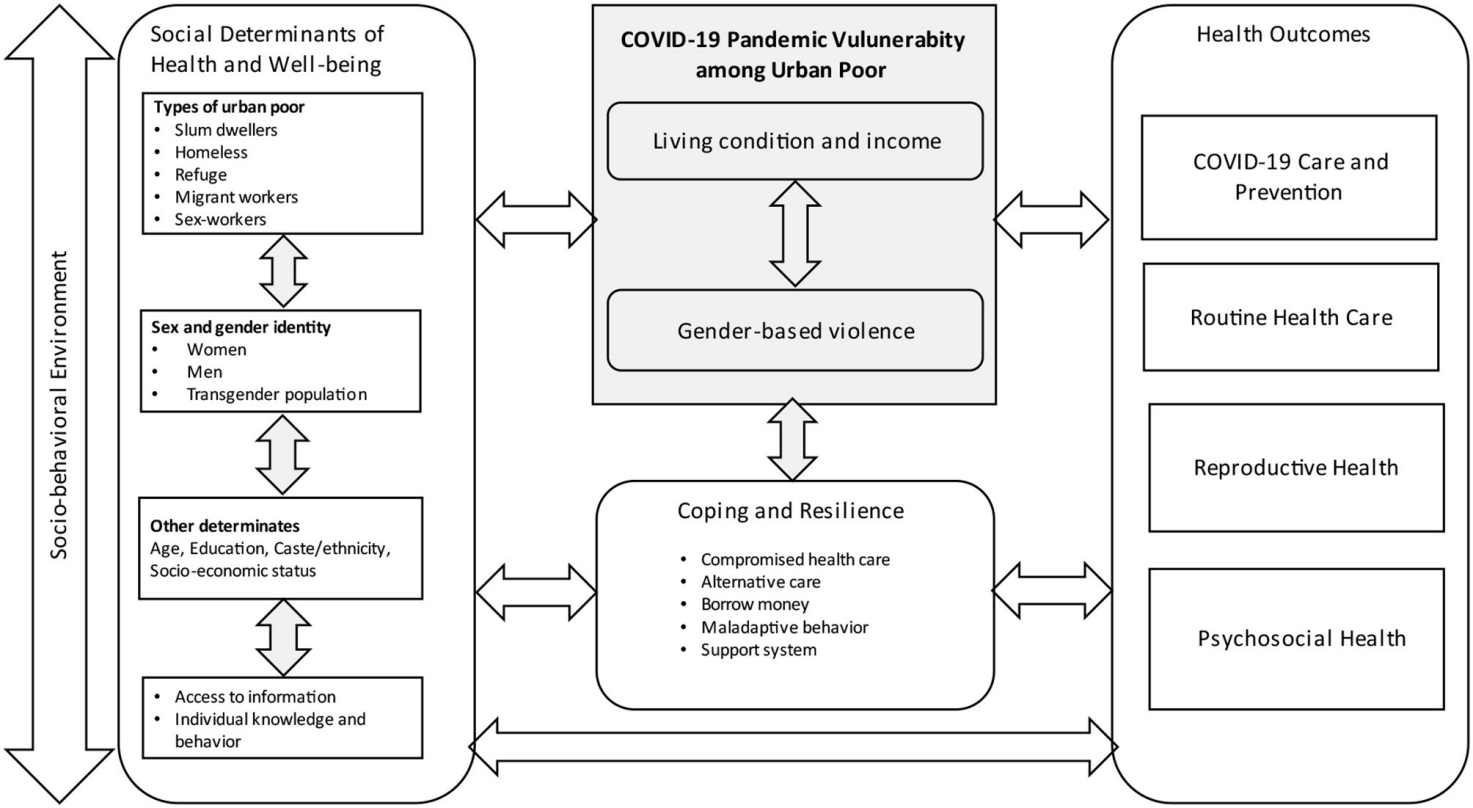
Conceptual framework on COVID-19 pandemic vulnerability among urban poor in low-and middle-income countries.

Figure 2 depicts our understanding of COVID-19 pandemic vulnerability among the urban poor in LMICs. The risk was often compounded by their poor living conditions, loss of income, and food insecurity—such deprivation frequently associated with gender-based violence, which affected health outcomes such as COVID-19 care and prevention, routine health care, reproductive health care health, and psychosocial health. Furthermore, the studies revealed a wide range of resilience strategies among urban poor. The impact of vulnerability was also influenced by four factors: the type of urban poor, gender, gender identity, other social determinants such as age, education, caste/ethnicity, and individual socioeconomic status.

### 3.1. Living condition, income, and food insecurity

During the pandemic, livelihood of many urban poor affected as public construction projects on shelters, roads, and drains were stopped. Street food and other vendors also stopped working (25, 26). Workers in many small companies and households help, mostly women, were laid off (27, 31, 35, 43). Women sex workers and the transgender population were loss their income (23, 25, 30). This resulted in food insecurity and limited health care expenditure. Discriminated income and fewer savings have increased women's vulnerability during the pandemic (26).

Around 80% of women-headed families experienced food scarcity compared to 20% of men-headed families (26, 59). In Dhaka, Bangladesh, 97% women and 74% men (49), Nairobi 77% women and 68% men (55), Nairobi, Kilifi, Kisumu 73% women (41), Delhi, India, 52% women and 82% men (42), in Colombo, Sri Lanka 67% women (50) reported food insecurity. Women faced more challenges than men due to gender inequity in employment during the pandemic. Studies showed 100% women and 50% men in Delhi, India (42), 76% women in Kenya (41), almost equal number of women and men (95%) in Dhaka, Bangladesh (49) reported job loss. A few women sold household belongings to meet their everyday needs, house rent and medical needs. For instance, a woman sold her child's bicycle to pay medical expenses (43). Girls were more likely than boys to share experiences of hunger in refugee camps (40). Income loss and inability to meet medical expenses were also seen in the transgender population (25).

One health volunteer promoting covid-appropriate behavior among slum dwellers noted that financial limitations forced them to pay for food and rent rather than buy gloves and sanitizers (34). One woman expressed, “we may survive the coronavirus, but we will most likely perish from hunger and depression” (27). Hunger, insecurity, and fear has contributed to a surge in violence experienced by women (26, 37, 39, 42). One woman explained, “when there is no food, and family members are requesting food, it is natural for men to become angry” (37). Young women saw men's role as that of “providers” which was crucial during economic insecurity. COVID-19-related income loss by men led to relationship conflicts and separation (41).

### 3.2. Gender-based violence

**Figure 3:**
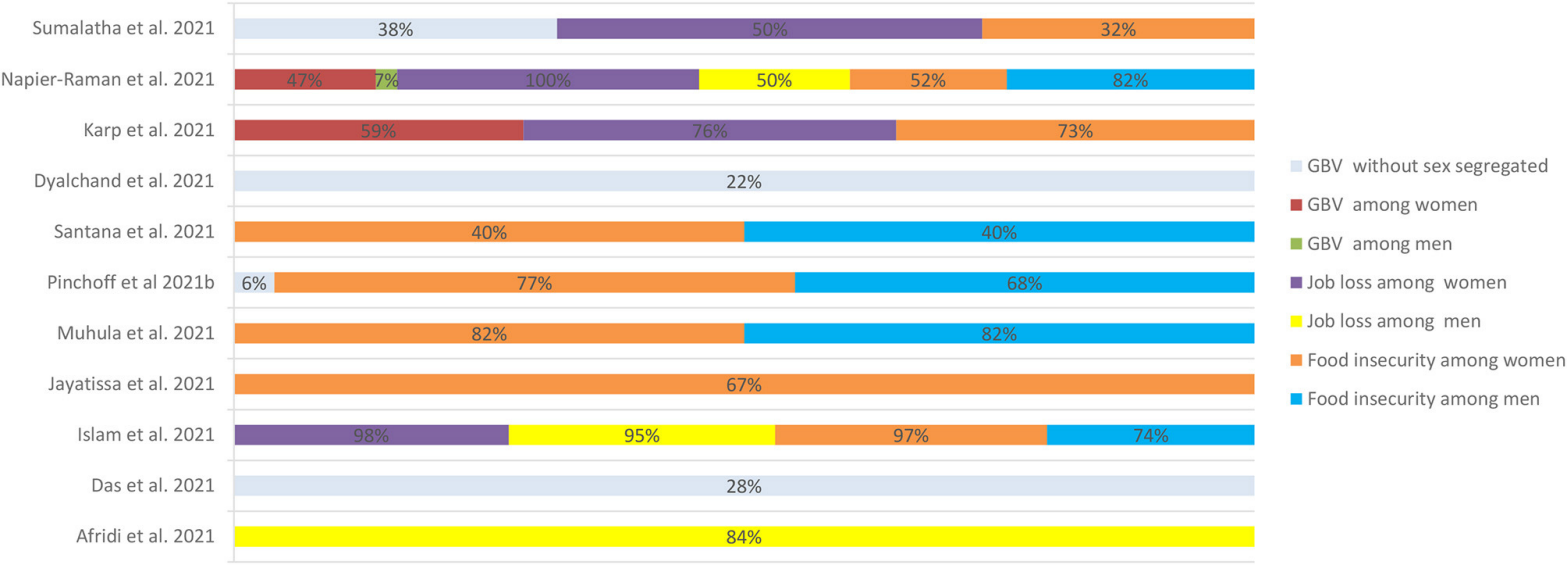
Loss of income, food insecurity and gender-based violence during COVID-19 among urban poor in low-and middle-income countries.

Above half of women reported experiencing violence during the COVID-19 pandemic and lockdowns. Gender-based violence (GBV) was recorded in 28% of urban poor women in West Bengal (48), 22% in Pune (39), 38% in Delhi, Mumbai, and Kochi (43), and 47% in Delhi, India (42). Similarly, 6.2% of Nairobi residents (55) and 59% of Nairobi, Kilifi, and Kisumu slum dwellers (41) in Kenya reported experiencing GBV. In many instances, the women were overworked in the home and endured intimate partner violence (28). All family members confined at home for extended periods, unemployment, and financial distress increased women's vulnerability to violence (31, 39, 43, 59). Married women reported experiencing more GBV, and women stated that food shortage strained their marital relationships (40). Women were abused when voicing concerns about food for their children. This strained their relationships and, at times, increased suicidal thoughts (24, 37, 43). Fear of infection has affected physical intimacy in relationships (41). The transgender population also reported verbal harassment from the public while out for work to avoid income loss (23, 25). Figure 3 presents the loss of income, food insecurity and gender-based violence during COVID-19 among urban poor in low-and middle-income countries.

### 3.3. Health outcomes

#### 3.3.1. COVID-19 prevention and care

Many urban poor did not possess sources of information like television or mobiles. They learnt about COVID-19 related information from watching television at neighborhood stores or tea stalls (40). Women, being more homebound, without access to mobile phones, requested neighbors or relatives to explain COVID-19 news updates (33). Similarly, the transgender population collected information on pandemic-appropriate behavior using their mobile phones (23, 25). Women were less affected by COVID-19 preventive restrictions as they usually spent more time indoors with domestic work. Compared to boys, girls adhered more to Covid guidelines and stayed indoors (40). A refugee woman expressed, 'the ladies frequently cook and serve meals to everyone, and their work remained largely unchanged' (40). Fear of COVID-19 prevented healthcare access for women and the vulnerable population (40, 59). Many affected women perceived isolation centers as “prisons” with meager facilities. Those migrating were labeled as “outsiders” and faced more difficulties (26, 28, 38). In some instances, women sex workers were viewed as COVID-19 carriers (30). On the other hand, transgender population in India expressed being subjected to the double discrimination of their low socioeconomic status and gender identity (24, 25). They had to wait for long periods to receive masks, soaps or medicines and even had difficulty during COVID testing (23, 25).

COVID-19 was active in 25% of women and 18% of men; the recovery rate was 25% for women and 22% for men, and the death rate was 3% for women and 5% for men (47). The median time to diagnose COVID-19 was 7 (IQR, 6.6–7.6) days for women and 8 (IQR, 7.5–8.6) days for men. Women had an average of 11 times direct contacts, while men had 16 times (56). Preventive practices avoiding COVID-19 (64%) (54) and willingness to receive vaccination (66%) among men and women were almost equally (45). Similarly, in Kolkata, 93% of women and 96% of men were infected with COVID-19 (54) and in Delhi, 83% of women and 97% of men were infected with COVID-19 (42).

Women were hesitant to participate in COVID-19 awareness initiatives (59) but the men-women engagement in non-governmental initiatives was ~1:10 in Bangladesh slums leading women-focused COVID-19 management (26, 37, 59). Similarly, in men-centric awareness initiatives, as in the Syrian refugee community, men possessed more accurate and comprehensive information (29).

#### 3.3.2. Routine health care

During COVID-19, most urban poor struggled to obtain services at public hospitals and were unable to afford private hospitals disrupting routine care. Restriction of movement and COVID-19 preventive measures by hospitals were prevailing and doctors maintained a safe distance due to COVID-19 fears (40). Around 28% of women were unable to receive routine healthcare in Delhi, Mumbai, Kochi (43), 38% in Pune, India (39). About women 11% and 5% of men were reported to be unable to access needed routine care in Nairobi, Kenya (55). A study of HIV patients living in informal communities in Kibera, Kenya, found a 56% decline in HIV care uptake (53). Women sex workers stopped the visits to sexual and reproductive health services for pre-or post-exposure prevention to reduce risks of HIV infection or to collect contraceptives (30). The transgender population, also faced similar challenges in obtaining care (24). They avoided reaching out to health facilities for their chronic illnesses, or for problems like alcohol withdrawal (24, 25). Some of them discontinued the services for anti-retroviral therapy (24). Though digital platforms for consultation were promoted, many urban poor, particularly women, lacked access to mobile phones and had little understanding of the notion of digital health, making virtual health services inaccessible (28).

#### 3.3.3. Reproductive health care

Many women struggled to access reproductive health services as most public hospitals were devoted to COVID-19 care. There was a shortage of emergency services—more than two-thirds had difficulty in accessing antenatal care, while the remaining received care from urban primary health centers. Few mothers chose to seek maternity care in private facilities, but private facilities increased their fees, making them unaffordable (32, 36, 39).

Only one-third of the women received diagnostic services. One-third of mothers were unable to vaccinate their children. Many mothers missed vaccinations for their children due to their husbands' refusal. During the lockdown, many pregnant women were deprived of prenatal care (42). The healthcare professionals indicated an increase in non-institutional deliveries. One of the reasons many urban poor women avoided medical treatments and check-ups were unclear information about COVID-19 testing among pregnant women and the associated costs (32). Many refugee women were denied admission to health facilities because they lack a National Health Insurance Fund (NHIF) card (32). Most of the women encountered difficulties in accessing contraceptives and sanitary pads due to medical facility closures, non-availability in slums, and inflated costs (39). Due to school closures and a lack of finances to meet their personal requirements, the COVID-19 has accelerated cohabitation, unintended pregnancies, and early marriages among the urban poor girl (41). The women sex workers explained that they could not afford contraception which associated with unintended pregnancy (30). Some refugee women reported even they deliver alongside the road (non-institutional delivery) due to their inability to afford private hospitals (32). Many of them expected the need of online counseling for their reproductive health care.

#### 3.3.4. Psychosocial health

**Figure 4:**
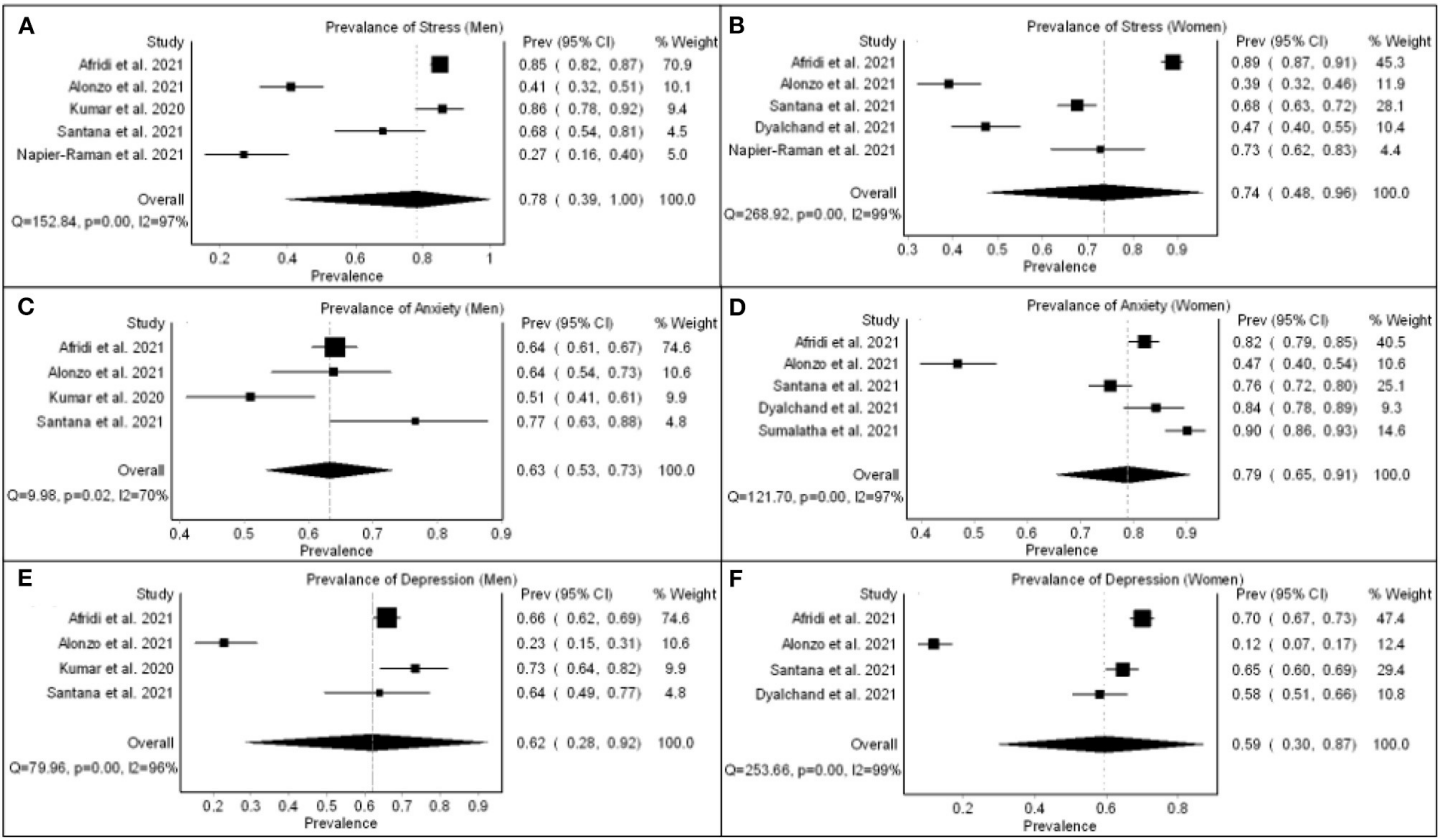
Pool prevalence of perceived mental health status - stress [**(A)** men and **(B)**] women, anxiety [**(C)** men and **(D)**] women, and depression [**(E)** men and **(F)** women] during COVID-19.

Figure 4 depicts the pool prevalence of perceived mental health status—stress, anxiety, and depression—among men and women during COVID-19. It was noticed that stress affects 74% of women and 78% of men, depression affects 59% of women and 62% of men, but anxiety affects 79% of women and 63% of men. In Dhaka, Bangladesh, the mean score for posttraumatic stress disorder was 15.4 (SD 3.0) for women and 14.9 (SD 3.7) for men (49). In Guatemala, burnout was reported by 12% of women, whereas 20% of men experienced burnout (46). Around 50% of women and 43% of men reported sleep disorders in Delhi (44). A nearly equal proportion of women and men (76%) reported sleep disorders in São Paulo, Brazil (57). A global online survey of refugees and migrants in LMICs revealed a significant relationship between gender and mental health outcomes, with men reporting lesser adverse effects of COVID-19 on their mental health outcomes than women (58).

Poor living conditions and economic hardships contributed to poor psychosocial health (34). Men had more stress than women during COVID-19. In the context of LMICs, they were preoccupied with the fear of job loss and financial insecurity, as they are primarily responsible for household sustenance. On the other hand, women, working or homemakers, had more anxiety than men, possibly because they are often the primary caregivers for vulnerable children and the elderly staying at home (41). They stayed in limited living space and felt they might transmit COVID infection to a dependent child or older adult, causing severe illness or even death (34). Men who worked for a living feared getting the infection and getting transferred to isolation centers (33). This fear was more in men than women due to more access to media information related to COVID-19 (32, 33, 36).

Women had burnout due to loss of job or an increase in household responsibilities (27, 38). In addition, all family members crowded together, and childcare added to women's stress (43). Transgender women and men in India with COVID-19 symptoms avoided health check-ups out of fear of stigma (24). Many expressed feelings of loneliness, social isolation, and sadness due to their movement restrictions. They were uneasy with social distance conventions because they were accustomed to living in groups (23, 25). The transgender population described being charged and yelled at by individuals who said that 'these hijra people are already contaminated and can transmit to us'. Sometimes they were not accepted at their rented residence (25). They fear being stigmatized for corona's rest of their lives, similar to HIV. Many claimed that social media outlets exacerbated their panic (25).

### 3.4. Coping and resilience

The urban poor adopted both healthy and unhealthy ways to cope with COVID-19 pandemic. With reduced opportunities for income among the urban poor, many women started modest businesses, such as tiffin stalls; others relied on government rations to feed their families while reducing their food consumption during the pandemics (27, 35). Women reduced expenses by substituting menstrual pads with clothes, not recharging mobile phones, and using alternatives to gas for cooking (28). Sometimes they borrowed money from money lenders at high rates to cover daily expenses (43).

In order to maintain COVID-19 preventive measures, most of them relied on government and non-government agencies for sanitizers and masks (34, 51). For instance, 'after groups ceased supplying free masks and soaps, 95% of inhabitants stopped using' them. Households purchased masks for men as they worked outside, whereas women managed using a scarf (37). Many urban poor preferred home remedies or continued their treatment at local pharmacies and informal healthcare practitioners (51, 52, 59). Some NGOs provided information and support for women groups affected by the pandemic, such as sex workers (30). Women managed their stress differently. Most slum residents kept themselves engrossed in prayer during their isolation (59). Some of them used the government tele-counseling service, but most were unaware of it (28).

## 4. Discussion

This systematic review describes sex and gender identity as potential social determinants of urban poor health during the COVID-19 pandemic. Many studies have found that food insecurity, access to care, and information via digital technology disproportionately affect women among the urban poor, as they always prioritize the needs of others. This review contributes to a better understanding of public health policy and planning among the urban poor during any emergency, including pandemics.

The gender is a social construct that refers to the responsibilities and expectations placed on men and women in a given society, roles that vary according to time, place, and life stage (60). According to WHO, gender refers to the socially constructed characteristics of men and women, such as norms, roles, and relationships among and between groups of men and women (61). Both urban poor men and women are affected by gender-related health issues (62). COVID-19 lockdowns enhance the probability of urban poor people in LMICs falling into extreme poverty. They faced dual burden of poverty and infection, which breaks their livelihood. The detrimental effect of gender appears to affect urban poor women during the COVID-19 pandemic. Anxiety among women was found to be higher in comparison to men. Women appear to incur more adverse health outcomes, primarily associated with gender disparities. In recent decades, government funding organizations and international organizations have prioritized research on women's health to address this inequity in policy and practices (61, 63, 64).

Economic instability, including food insecurity and income, varies by the types of urban poor such as slum dwellers, homeless, refugee, migrant workers, commercial sex workers, gender identity—men, women, and transgender population and level of education (18, 65). While there has been increasing recognition of the social inequalities experienced by the urban poor, there is hardly any common strategies to address the issues (66, 67). There is a lack of comprehensive understanding and adequate information regarding the common context-specific challenges in terms of gender (18). Thus, this review indicates the need for further primary research on the gender dimension of health challenges among urban poor during any emergency situations for urban policy and planning.

Universal access to essential health care services is critical for achieving the Sustainable Development Goals (SDGs) (63, 68). Access to primary, specialty, and emergency care and affordable cost, health literacy, and the quality of care are integral components of essential health services (69). There is a lack of primary care services and less insurance among the urban poor. Effective service design requires intersectoral collaboration and community-centered health care service planning (69, 70). Moreover, improving access to and the quality of care in vulnerable populations will require developing novel strategies for incorporating limited resources and transforming care to meet the needs of changing communities (64). Collaboration for integration and transformation is more critical than ever to establish relationships with a diverse group of community stakeholders to comprehend and meet the needs of the urban poor (69, 71). Thus, the built environment—improved housing, increased food access, and elimination of all forms of discrimination and violence—is critical for urban health policy and planning.

Healthcare data should become a policy priority for urban public health (72, 73). Digital platforms were created during COVID-19 to track case numbers, hospital bed availability, and community literacy. However, the study's findings revealed disparities in access to technological outcomes between men and women in slum settings. The differences in technology outcomes between gender identities revealed disparate predictors of mobile phone ownership, internet access, and text messaging among men, women, and the transgender population. As a result, they were noticed to have higher technological awareness and utilization (8). It is also likely that such slum residences closer to wealthy neighborhoods will have lower crime and poverty rates, which are significant predictors of gender disparities in access to technology. However, our systematic review indicates that women's access to technology requires further exploration.

The findings indicate that improving household education is critical for addressing disparities in women's access to and use of mobile phones, the internet, and text messaging in slum settings, owing to the consistency of household educational level as a predictor across all of these technology indicators (10, 71). This condition may be explained because women are more likely to rely on men in their households for financial support (6, 43). The absence of a wage earner in the family can substantially impact the household's financial wellbeing (39, 70). Hence, women's education is a critical predictor of all technology results in slum areas (5, 68–76). Gender disparities, in particular, have been attributed to conventions such as a lack of emphasis on women's education and financial independence.

We found only three qualitative studies on the non-binary gender population in the COVID-19 context in LMICs, which indicates the need for more research among these populations. To the extent that this paper seeks to describe pooled estimates of risk, these features may be viewed as potential limitations because there are not enough studies for risk estimations, indicating the need for additional research in these domains. In addition, quantitative studies on social and cultural beliefs prior to, during, and after the pandemic are critical in the context of LMICs. Along with women's education, governments and non-governmental organizations should develop gender streaming policies that include plans for changing stereotypical and culturally rooted gender division attitudes in societies. Furthermore, the findings show that in some areas, men are more disadvantaged than women. Hence, when generalizations about women being more vulnerable than men are made, caution should be exercised. Policies should be based on a gender perspective so that both men and women are treated based on their circumstances when necessary. As a result, primary quantitative data based on gender is critical for gender-sensitive health planning, particularly among the urban poor.

## 5. Conclusion

This review highlights that abolishing all forms of discrimination and violence in income opportunities among women, men, and other gender identities is critical for health policy and planning for the urban poor. However, sex and gender identity were frequently influence health and wellbeing among the urban poor. However, there is limited information on sex-segregated data or studies on the gender dimension of health among the urban poor during any emergency, including the COVID-19 pandemic. This review emphasizes the critical importance of conducting future research on the gender dimensions of health among the urban poor during emergencies. While the severity of the pandemic varies by sex and gender identity, their vulnerability also varies by caste/ethnicity, literacy, and economic status, which suggests the importance of focusing on how and why gender intersects with other social variables under structural conditions of disadvantages and discriminations. The findings also imply that in-depth research on emergency vulnerability among the urban poor is required—a broad range social determinants and their intersections that influence their health and disease experience.

## Data availability statement

The original contributions presented in the study are included in the article/Supplementary material, further inquiries can be directed to the corresponding author.

## Author contributions

KCS, SN, KS, and PM developed the protocol. SN, SD, and MS completed the search, screened the articles for inclusion, and extracted the data. KCS, SN, PM, KS, and GD extracted the data and synthesized the findings, interpreted the results, and drafted the manuscript. SN and KS completed the risk of bias assessments. SPP, SP, and MO interpreted the results. All authors critically revised the manuscript and approved the final version.

## References

[1] United Nations Department Department of economic and social affairs Population division. 68% of the world population projected to live in urban areas by 2050, says UN. 2018 *Revision of World Urbanisation Prospects* (2018).

[2] McMichael AJ. The urban environment and health in a world of increasing globalization: issues for developing countries. Bull World Health Organ. (2000) 78:1117–26. 11019460 PMC2560839

[3] World Health Organization. Regional Office for South-East Asia. Addressing health of the urban poor in South-East Asia Region: challenges and opportunities. WHO Regional Office for South-East Asia. (2011). Available online at: https://apps.who.int/iris/handle/10665/204753 (accessed April 23, 2022).

[4] Sclar ED Garau P Carolini G. The 21st century health challenge of slums and cities. Lancet. (2005) 365:901–3. 10.1016/S0140-6736(05)71049-7 15752535

[5] Rao KD Peters DH. Urban health in India: many challenges, few solutions. Lancet Glob Health. (2015) 3:e729–30. 10.1016/S2214-109X(15)00210-7 26566742

[6] Afsana K Wahid SS. Health care for poor people in the urban slums of Bangladesh. Lancet. (2013) 382:2049–51. 10.1016/S0140-6736(13)62295-3 24268606

[7] Battersby J McLachlan M. Urban food insecurity: a neglected public health challenge. S Afr Med J. (2013) 103:716–7. 10.7196/SAMJ.7463 24079620

[8] van de Vijver S Oti S Oduor C Ezeh A Lange J Agyemang C . Challenges of health programmes in slums. Lancet. (2015) 386:2114–6. 10.1016/S0140-6736(15)00385-2 26452707

[9] Shetty P. Health care for urban poor falls through the gap. Lancet. (2011) 377:627–8. 10.1016/S0140-6736(11)60215-8

[10] Hone T Macinko J Millett C. Revisiting alma-ata: what is the role of primary health care in achieving the sustainable development goals? The Lancet. (2018) 392:1461–72. 10.1016/S0140-6736(18)31829-4 30343860

[11] Sachs JD Karim SA Aknin L. Lancet COVID-19 commissioners, task force chairs, and commission secretariat. Lancet COVID-19 Commission statement on the occasion of the 75th session of the UN general assembly. Lancet. (2020) 396:1102–24. 10.1016/S0140-6736(20)31927-9 32941825 PMC7489891

[12] Tampe T. Potential impacts of COVID-19 in urban slums: addressing challenges to protect the world's most vulnerable. Cities Health. (2020) 28:1–4. 10.1080/23748834.2020.1791443

[13] Bukhman G Mocumbi AO Atun R Becker AE Bhutta Z Binagwaho A . The lancet NCDI poverty commission: bridging a gap in universal health coverage for the poorest billion. The Lancet. (2020) 396:991–1044. 10.1016/S0140-6736(20)31907-3 32941823 PMC7489932

[14] Dlamini NJ. Gender-based violence, twin pandemic to COVID-19. Crit Sociol. (2021) 47:583–90. 10.1177/0896920520975465 38603014 PMC7723732

[15] Sahoo KC Negi S Patel K Mishra BK Palo SK Pati S. Challenges in maternal and child health services delivery and access during pandemics or public health disasters in low-and middle-income countries: a systematic review. InHealthcare. (2021) 9:828. 10.3390/healthcare9070828 34209238 PMC8306470

[16] Jacob CM Briana DD Di Renzo GC Modi N Bustreo F Conti G . Building resilient societies after COVID-19: the case for investing in maternal, neonatal, and child health. Lancet Public Health. (2020) 5:e624–7. 10.1016/S2468-2667(20)30200-0 32971008 PMC7505549

[17] Chew M Das P Aujla M Horton R. Advancing racial and ethnic equity in science, medicine, and health: a call for papers. Lancet. (2021) 398:1287–9. 10.1016/S0140-6736(21)02095-X 34592136

[18] Abdi F Mahmoodi Z Afsahi F Shaterian N Rahnemaei FA. Social determinants of domestic violence against suburban women in developing countries: a systematic review. Obstet Gynecol Sci. (2021) 64:131–42. 10.5468/ogs.20211 33503736 PMC7991000

[19] Abdi F Rahnemaei FA Shojaei P Afsahi F Mahmoodi Z. Social determinants of mental health of women living in slum: a systematic review. Obstet Gynecol Sci. (2021) 64:143–55. 10.5468/ogs.20264 33685034 PMC7990997

[20] Page MJ McKenzie JE Bossuyt PM Boutron I Hoffmann TC Mulrow CD . The PRISMA 2020 statement: an updated guideline for reporting systematic reviews. Syst rev. (2021) 10:1–1. 10.1186/s13643-021-01626-4 33781348 PMC8008539

[21] Srivastava A Thomson SB. Framework analysis: a qualitative methodology for applied policy research. JOAAG. (2009) 4:72–9. Available online at: https://ssrn.com/abstract=276070526169286

[22] Hong QN Fàbregues S Bartlett G. The mixed methods appraisal tool (MMAT) version 2018 for information professionals and researchers. Educ Inf . (2018) 34:285–91. 10.3233/EFI-18022129132909

[23] Banerjee D Rao TS. “The Graying Minority”: lived experiences and psychosocial challenges of older transgender adults during the COVID-19 pandemic in India, a qualitative exploration. Front Psych. (2021) 3:1510. 10.3389/fpsyt.2020.604472 33488427 PMC7820119

[24] Pandya A Redcay A. Impact of COVID-19 on transgender women and Hijra: insights from Gujarat, India. J Hum Rights Soc Work. (2021) 19:1–0. 10.21203/rs.3.rs-44619/v1 34307834 PMC8287106

[25] Jalil T Rahman MM Rahman M Rashid SF. On the fringes: impact of COVID-19 shutdown on the mental health condition of the Hijra community: a qualitative study conducted in Dhaka, Bangladesh. Ment Health. (2021) 1–11. Available online at: https://new.bracjpgsph.org/public/assets/front/jpgsph/pdf/covid/research/pdf/RHRN-MENTAL-HEALTH-11-March-2021.pdf

[26] Akter S Dhar TK Rahman AI Uddin MK. Investigating the resilience of refugee camps to COVID-19: a case of Rohingya settlements in Bangladesh. J Migr Health. (2021) 4:100052. 10.1016/j.jmh.2021.100052 34405195 PMC8352112

[27] Arora S Majumder M. Where is My Home? Gendered precarity and the experience of Covid-19 among women migrant workers from delhi and national capital Region, India. Gend Work Organ. (2021) 3:12700. 10.1111/gwao.12700 34219998 PMC8239831

[28] Azeez EP A Negi DP Rani A AP SK. The impact of COVID-19 on migrant women workers in India. Eurasian Geogr Econ. (2021) 62:93–112. 10.1080/15387216.2020.1843513

[29] Douedari Y Alhaffar M Al-Twaish M Mkhallalati H. “Ten years of war! You expect people to fear a ‘germ'?” A qualitative study of initial perceptions and responses to the COVID-19 pandemic among displaced communities in opposition-controlled northwest. Syria J migr health. (2020) 1:100021. 10.1016/j.jmh.2020.100021 33458715 PMC7790454

[30] Gichuna S Hassan R Sanders T Campbell R Mutonyi M Mwangi P. Access to healthcare in a time of COVID-19: sex workers in crisis in Nairobi, Kenya. Glob Public Health. (2020) 15:1430–42. 10.1080/17441692.2020.1810298 32816628

[31] Kar S Mohapatra I Mishra A Banerjee A. Mitigation strategies and Covid appropriate and risk behavior: a descriptive study at slums of Bhubaneswar, Odisha. Adv Res J Multidisc Disco. (2021) 57:01–6. 10.5281/zenodo.4774482

[32] Lusambili AM Martini M Abdirahman F Asante A . “We have a lot of home deliveries” A qualitative study on the impact of COVID-19 on access to and utilization of reproductive, maternal, newborn and child health care among refugee women in urban Eastleigh, Kenya. J Migr Health. (2020) 1:100025. 10.1016/j.jmh.2020.100025 34405176 PMC8352096

[33] Mathias K Rawat M Philip S Grills N. We've got through hard times before: acute mental distress and coping among disadvantaged groups during COVID-19 lockdown in North India-a qualitative study. Int J equity health. (2020) 19:1–2. 10.1186/s12939-020-01345-7 33334344 PMC7745174

[34] Munajed D Ekren E. Exploring the impact of multidimensional refugee vulnerability on distancing as a protective measure against COVID-19: The case of Syrian refugees in Lebanon and Turkey. J Migr Health. (2020) 1:1. 10.1016/j.jmh.2020.100023 34405174 PMC8352139

[35] Nanda J. Impact of Lockdown for COVID-19 on Female Domestic Workers: A Case Study from Cuttack City. Available at SSRN 3628346 2020. 10.2139/ssrn.3628346

[36] Oluoch-Aridi J Chelagat T Nyikuri MM . COVID-19 Effect on access to maternal health services in Kenya. Front Glob Women's Health. (2020) 1:19. 10.3389/fgwh.2020.599267 34816169 PMC8593959

[37] Rashid SF Aktar B Farnaz N Theobald S Ali S Alam W . Fault-lines in the public health approach to COVID-19: recognizing inequities and ground realities of poor residents lives in the slums of Dhaka City, Bangladesh. Soc Sci Human. (2020) 3:8577. 10.2139/ssrn.3608577

[38] Zakar R Yousaf F Zakar MZ Fischer F. Sociocultural challenges in the implementation of COVID-19 public health measures: Results from a qualitative study in Punjab, Pakistan. Front Public Health. (2021) 9:3825. 10.3389/fpubh.2021.703825 34354975 PMC8329025

[39] Dyalchand A. Impact of COVID-19 pandemic on health care, essential services, employment, and economic activities, mental distress, and perceived quality of life among women and girls. Bharati Vidyapeeth Med J. (2021) 2:21–9. 10.56136/BVMJ/2021_00024

[40] Guglielmi S Seager J Mitu K Baird S Jones N. Exploring the impacts of COVID-19 on Rohingya adolescents in Cox's Bazar: a mixed-methods study. J Migr Health. (2020) 1:100031. 10.1016/j.jmh.2020.100031 34405179 PMC8352087

[41] Karp C Moreau C Sheehy G Anjur-Dietrich S Mbushi F Muluve E . Youth relationships in the Era of COVID-19: a mixed-methods study among adolescent girls and young women in Kenya. J Adolesc Health. (2021) 69:754–61. 10.1016/j.jadohealth.2021.07.017 34465510 PMC8460286

[42] Napier-Raman S Rattani A Qaiyum Y Bose V Seth R Raman S. Impact of COVID-19 on the lives of vulnerable young people in New Delhi, India: a mixed method study. BMJ Paediatr Open. (2021) 5:1171. 10.1136/bmjpo-2021-001171 34345717 PMC8316697

[43] Sumalatha BS Bhat LD Chitra KP. Impact of Covid-19 on informal sector: a study of women domestic workers in India. Indian J Econ. (2021) 69:441–61. 10.1177/0019466221102384535942415

[44] Afridi F Dhillon A Roy S. The gendered crisis: livelihoods and mental well-being in India during COVID-19. World Inst Develop Econ Res (UNU-WIDER). (2021). 10.35188/UNU-WIDER/2021/003-035112530

[45] Aguilar Ticona JP Nery N. Victoriano R. Willingness to get the COVID-19 vaccine among residents of slum settlements. Vaccines. (2021) 9:951. 10.3390/vaccines9090951 34579188 PMC8472908

[46] Alonzo D Popescu M Zubaroglu-Ioannides P. The current pandemic, a complex emergency? Mental health impact of the COVID-19 pandemic on highly vulnerable communities in Guatemala. Int J Soc Psych. (2021) 3:00207640211027212. 10.1177/00207640211027212 34154427 PMC8225464

[47] Cobre AD Böger B Fachi MM Vilhena RD Domingos EL Tonin FS . Risk factors associated with delay in diagnosis and mortality in patients with COVID-19 in the city of Rio de Janeiro, Brazil. Cienc saude colet. (2020) 25:4131–40. 10.1590/1413-812320202510.2.26882020 33027349

[48] Das T Roy TB Roy R. Reintegration with family and intimate partner violence (IPV) against women among the returnee migrant worker's family during COVID-19 induced lockdown: a Block-level analysis using multinomial logistic regression model. Child Youth Serv Rev. (2021) 130:106226. 10.1016/j.childyouth.2021.106226 34511676 PMC8416023

[49] Islam MS Rahman ME Banik R Emran MGI Saiara N Hossain S . Financial and mental health concerns of impoverished urban-dwelling Bangladeshi people during COVID-19. Front Psychol. (2021) 3:3326. 10.3389/fpsyg.2021.663687 34421719 PMC8377359

[50] Jayatissa R Herath HP Perera AG Dayaratne TT De Alwis ND Nanayakkara HP. Impact of COVID-19 on child malnutrition, obesity in women and household food insecurity in underserved urban settlements in Sri Lanka: a prospective follow-up study. Public Health Nutr. (2021) 3:1–9. 10.1017/S1368980021001841 33902778 PMC8144823

[51] Kumar K Mehra A Sahoo S Nehra R Grover S. The psychological impact of COVID-19 pandemic and lockdown on the migrant workers: a cross-sectional survey. Asian J Psych. (2020) 53:102252. 10.1016/j.ajp.2020.102252 32593970 PMC7305726

[52] Mamun M Fatima K. Slum Dwellers' perception about COVID-19: A Study in Dhaka Metropolis Slums. Technium Soc Sci J. (2021) 21:728. 10.47577/tssj.v21i1.3797

[53] Muhula S Opanga Y Oramisi V Ngugi C Ngunu C Carter J . Impact of the first wave of the COVID-19 pandemic on HIV/AIDS programming in Kenya: evidence from Kibera informal settlement and COVID-19 hotspot counties. Int J Environ Res Public Health. (2021) 18:6009. 10.3390/ijerph18116009 34205036 PMC8199875

[54] Mukhopadhyay J. Optimism under the holocaust of COVID-19 in Kolkata slum. J Community Health Manage. (2020) 7:44–50. 10.18231/j.jchm.2020.021

[55] Pinchoff J Austrian K Rajshekhar N Abuya T Kangwana B Ochako R . Gendered economic, social and health effects of the COVID-19 pandemic and mitigation policies in Kenya: evidence from a prospective cohort survey in Nairobi informal settlements. BMJ open. (2021) 11:e042749. 10.1136/bmjopen-2020-042749 33658260 PMC7931215

[56] Quaife M van Zandvoort K Gimma A Shah K McCreesh N Prem K . The impact of COVID-19 control measures on social contacts and transmission in Kenyan informal settlements. BMC Med. (2020) 18:1–1. 10.1186/s12916-020-01779-4 33012285 PMC7533154

[57] Santana CLA Manfrinato CV Souza PRP Marino A Condé VF Stedefeldt E . Psychological distress, low-income, and socio-economic vulnerability in the COVID-19 pandemic. Public Health. (2021) 199:42–5. 10.1016/j.puhe.2021.08.016 34537575 PMC8390360

[58] Spiritus-Beerden E Verelst A Devlieger I Primdahl NL Guedes FB Chiarenza A . Mental health of refugees and migrants during the COVID-19 pandemic: the role of experienced discrimination and daily stressors. Int J of Environ Res Public Health. (2021) 18:6354. 10.3390/ijerph18126354 34208243 PMC8296172

[59] Akter S Hakim SS Rahman MS. Planning for Pandemic Resilience: COVID-19 experience from urban slums in Khulna, Bangladesh. J Urban Manag. (2021) 10:325–44. 10.1016/j.jum.2021.08.003

[60] Phillips SP. Defining and measuring gender: a social determinant of health whose time has come. Int J Equity Health. (2005) 4:1–4. 10.1186/1475-9276-4-11 16014164 PMC1180842

[61] World Health Organization Gender and health. World Health Organization (WHO). (2021). Available online at: https://www.who.int/health-topics/gender#tab=tab_1 (accessed April 23, 2022).

[62] Connor J Madhavan S Mokashi M Amanuel H Johnson NR Pace LE . Health risks and outcomes that disproportionately affect women during the Covid-19 pandemic: a review. Soc Sci Med. (2020) 266:113364. 10.1016/j.socscimed.2020.113364 32950924 PMC7487147

[63] Royston G Pakenham-Walsh N Zielinski C. Universal access to essential health information: accelerating progress towards universal health coverage and other SDG health targets. BMJ Glob Health. (2020) 5:e002475. 10.1136/bmjgh-2020-002475 32424012 PMC7245367

[64] Warren CE Bellows B Marcus R Downey J Kennedy S Kureshy N. Strength in diversity: integrating community in primary health care to advance universal health coverage. Glob Health, Sci Prac. (2021) 9(Supplement 1):S1–5. 10.9745/GHSP-D-21-00125 33727314 PMC7971373

[65] Salgado M Madureira J Mendes AS Torres A Teixeira JP Oliveira MD. Environmental determinants of population health in urban settings. A systematic review. BMC Public Health. (2020) 20:1–1. 10.1186/s12889-020-08905-0 32493328 PMC7271472

[66] Butcher S Cociña C Yap C Levy C. Localizing the Sustainable Development Goals: An Urban Equality Perspective. International Engagement Brief #2. London: Knowledge in Action for Urban Equality. The Bartlett Development Planning Unit (2021).

[67] Preker AS Cotlear D Kwon S Atun R Avila C. Universal health care in middle-income countries: Lessons from four countries. J Glob Health. (2021) 11:4. 10.7189/jogh.11.16004 34912557 PMC8645236

[68] Chapman AR. Assessing the universal health coverage target in the sustainable development goals from a human rights perspective. BMC Int Health Hum Rights. (2016) 16:1–9. 10.1186/s12914-016-0106-y 27978827 PMC5159947

[69] Bhatt J Bathija P. Ensuring access to quality health care in vulnerable communities. Acad med. (*2018*) 93:1271. 10.1097/ACM.0000000000002254 29697433 PMC6112847

[70] Aletta F Oberman T Kang J. Positive health-related effects of perceiving urban soundscapes: a systematic review. Lancet. (2018) 392:S3. 10.1016/S0140-6736(18)32044-0 30380601 PMC6266166

[71] Fenner R Cernev T. The implications of the COVID-19 pandemic for delivering the sustainable development goals. Futures. (2021) 128:102726. 10.1016/j.futures.2021.102726 34658398 PMC8510889

[72] Labrique AB Wadhwani C Williams KA Lamptey P Hesp C Luk R . Best practices in scaling digital health in low- and middle-income countries. Glob health. (2018) 14:1–8. 10.1186/s12992-018-0424-z 30390686 PMC6215624

[73] Joshi A Malhotra B Amadi C Loomba M Misra A Sharma S . Gender and the digital divide across urban slums of New Delhi, India: cross-sectional study. J med Internet Res. (2020) 22:e14714. 10.2196/14714 32343670 PMC7338923

[74] Sahoo KC Dubey S Dash GC Sahoo RK Sahay MR Negi S . A systematic review of water, sanitation, and hygiene for urban poor in low-and middle-income countries during the COVID-19 pandemic through a gendered lens. Int J Environ Res Public Health. (2022) 19:11845. 10.3390/ijerph191911845 36231147 PMC9565771

[75] Sahoo KC Sahay MR Dubey S Nayak S Negi S Mahapatra P . Community engagement and involvement in managing the COVID-19 pandemic among urban poor in low-and middle-income countries: a systematic scoping review and stakeholders mapping. Glob Health Action. (2023) 16:2133723. 10.1080/16549716.2022.2133723 36537837 PMC9769144

[76] Nunes NR Rodriguez A Cinacchi GB. Health and social care inequalities: the impact of COVID-19 on people experiencing homelessness in Brazil. Int J Environ Res Public Health. (2021) 18:5545. 10.3390/ijerph18115545 34067316 PMC8196886

