# Supplementary material for: Gender dimensions of health-related challenges among urban poor during COVID-19 pandemic in low-and middle-income countries: a systematic review and gap analysis
Source: Front Public Health. 2023 Jun 9;11:1170386. doi: 10.3389/fpubh.2023.1170386 (PMC10288984; doi:10.3389/fpubh.2023.1170386)
Supplement: Supplementary file 1 [file Data_Sheet_1.docx]

# Supplementary Material

**Appendix I**

**Search Terms**

**Database 1: PubMed**

| **PubMed** | **As on Date: 31st Aug 2021** |  |
| --- | --- | --- |
|  | **Query** | **Items found** |
| #5 | #1 AND #2 AND #3 (COVID-19 & SLUM &LMICs)(filter: 2019-2021) | 1258 |
| #4 | #1 AND #2 (COVID-19 & SLUM) | 5794 |
| #1  COVID-19 | "Pandemics" OR "COVID-19" OR "Novel corona virus" OR "Novel coronavirus" OR "COVID-19" OR "SARS-CoV-2" OR "Coronavirus" OR "COVID-19" OR COVID OR "19 Virus Disease*" OR "COVID-19 Virus Infection*" OR "2019-nCoV Infection*" OR "nCoV*" OR "Coronavirus Disease-19" OR "2019 Novel Coronavirus Disease" OR "2019 Novel Coronavirus Infection" OR "2019-nCoV Disease" OR "2019 nCoV Disease" OR 2019-nCoV* OR COVID19* OR "Coronavirus Disease 2019" OR Coronavirus* OR SARS* OR "SARS-CoV-2 Infection" OR "Infection, SARS-CoV-2" OR "SARS CoV 2 Infection " OR SARS-CoV-2* OR "COVID 19 Pandemic" | 203,581 |
| #2  SLUM | Slum* OR underprivileged OR “Informal settlements” OR “Urban informal settlement” OR Urban areas OR Urban poor OR settlements OR vulnerable places OR “Poverty Area*” OR Poverty OR Ghetto OR Jhugg* OR Rurban OR Refugee* OR Migrant* OR Transient* OR “Disadvantaged group” | 562,545 |
| #3  LMICs | "Africa" OR "Central America" OR "Afghanistan" OR "Armenia" OR "Bangladesh" OR "Bhutan" OR "Bolivia" OR "Cambodia" OR "Comoros" OR "Georgia Republic" OR "Guyana" OR "Haiti" OR "India* " OR "Indonesia" OR "Micronesia" OR "Kosovo" OR "Kyrgyzstan" OR "Laos" OR "Madagascar" OR "Moldova" OR "Mongolia" OR "Myanmar" OR "Nepal" OR "Pakistan" OR "Paraguay" OR "Philippines" OR "Samoa" OR "Melanesia" OR "Sri Lanka" OR "Syria" OR "Tajikistan" OR "Ukraine" OR "Uzbekistan" OR "Vanuatu" OR "Vietnam" OR "Yemen" OR "Korea" OR algeria OR angola OR benin OR botswana OR burkinafaso OR burundi OR Cameroon OR cape verde OR central african republic OR chad OR comoros OR "cote d'ivoire" OR ivory coast OR congo OR zaire OR Djibouti OR egypt OR equatorial guinea OR ethiopia OR eritrea OR gabon OR gambia OR ghana OR guinea OR guinee-bissau OR kenya OR Lesotho OR Liberia OR Libya OR Malawi OR Mali OR Mauritania OR Mauritius OR Mayotte OR Morocco OR Mozambique OR namibia OR niger OR nigeria OR reunion OR rwanda OR sahara OR saint Helena OR sao tome OR senegal OR seychelles OR sierraleone OR somalia OR south africa OR sudan OR swaziland OR togo OR tanzania OR tunisia OR uganda OR zambia OR zimbabwe OR georgia OR "solomon islands" OR "west bank" OR "gaza" OR kiribati OR "El Salvador" OR "caboverde" OR guatemala OR honduras OR nicaragua OR korea OR "kyrgyz" OR laos OR "low resource" OR "Under-resourced" OR "resource poor" OR "under-developed" OR "underdeveloped" OR "Developing country" OR "Developing countries" OR "Developing world" OR "Third world" OR lmic OR "low and middle income countries" OR LMICs | 2,642,789 |

**Database 2: Embase**

| **Embase** | **As on Date: 31st Aug 2021** |  |
| --- | --- | --- |
|  | **Query** | **Items found** |
| #5 | #1 AND #2 AND #3 (COVID-19 & SLUM & LMICs) | 385 |
| #4 | #1 AND #2 (COVID-19 & SLUM) | 1,195 |
| #1  COVID-19 | 'coronavirus disease 2019'/exp OR 'coronavirus disease 2019' OR 'severe acute respiratory syndrome coronavirus 2'/exp OR 'severe acute respiratory syndrome coronavirus 2' OR 'sars-cov-2 vaccine'/exp OR 'sars-cov-2 vaccine' | 155,105 |
| #2  SLUM | slum OR underprivileged OR 'informal settlements' OR 'urban areas' OR 'urban poor' OR settlements OR 'vulnerable places' OR 'poverty areas*' OR poverty OR 'areas, poverty' OR 'poverty area' OR slum* OR ghetto* OR 'slum area' | [104,8](https://www.embase.com/)99 |
| #3  LMICs | 'developing country'/exp OR 'Africa'/exp OR 'Central America'/exp OR 'Afghanistan'/exp OR 'Armenia'/exp OR 'Bangladesh'/exp OR 'Bhutan'/exp OR 'Bolivia'/exp OR 'Cambodia'/exp OR 'Comoros'/exp OR 'Georgia (republic)'/exp OR 'Guyana'/exp OR 'Haiti'/exp OR 'India'/exp OR 'Indonesia'/exp OR 'Federated States of Micronesia'/exp OR 'North Korea'/exp OR 'Kosovo'/exp OR 'Kyrgyzstan'/exp OR 'Laos'/exp OR 'Madagascar'/exp OR 'Moldova'/exp OR 'Mongolia'/exp OR 'Myanmar'/exp OR 'Nepal'/exp OR 'Pakistan'/exp OR 'Papua New Guinea'/exp OR 'Paraguay'/exp OR 'Philippines'/exp OR 'Samoan IslORs'/exp OR 'Melanesia'/exp OR 'Sri Lanka'/exp OR 'Syrian Arab Republic'/exp OR 'Tajikistan'/exp OR 'Timor-Leste'/exp OR 'Ukraine'/exp OR 'Uzbekistan'/exp OR 'Vanuatu'/exp OR 'Viet Nam'/exp OR 'Yemen'/exp OR ‘Afghanistan’:ab,ti OR ‘Armenia’:ab,ti OR ‘Bangladesh’:ab,ti OR ‘Bhutan’:ab,ti OR ‘Bolivia’:ab,ti OR ‘Cambodia’:ab,ti OR ‘Comoros’:ab,ti OR ‘Georgia’:ab,ti OR ‘Guyana’:ab,ti OR ‘Haiti’:ab,ti OR ‘India’:ab,ti OR ‘Indonesia’:ab,ti OR ‘Micronesia’:ab,ti OR ‘Korea’:ab,ti OR ‘Kosovo’:ab,ti OR ‘Kyrgyzstan’:ab,ti OR ‘Laos’:ab,ti OR ‘Madagascar’:ab,ti OR Micronesia:ab,ti OR ‘Moldova’:ab,ti OR ‘Mongolia’:ab,ti OR ‘Myanmar’:ab,ti OR ‘Nepal’:ab,ti OR ‘Pakistan’:ab,ti OR ‘Papua New Guinea’:ab,ti OR ‘Paraguay’:ab,ti OR ‘Philippines’:ab,ti OR ‘Samoa’:ab,ti OR ‘Melanesia’:ab,ti OR ‘Sri Lanka’:ab,ti OR ‘Syria’:ab,ti OR ‘Tajikistan’:ab,ti OR ‘East Timor’:ab,ti OR ‘Ukraine’:ab,ti OR ‘Uzbekistan’:ab,ti OR ‘Vanuatu’:ab,ti OR ‘Vietnam’:ab,ti OR ‘Yemen’:ab,ti OR Africa:ab,ti OR African:ab,ti OR algeria:ab,ti OR angola:ab,ti OR benin:ab,ti OR botswana:ab,ti OR ‘burkinafaso’:ab,ti OR burundi:ab,ti OR cameroon:ab,ti OR ‘cape verde’:ab,ti OR ‘central african republic’:ab,ti OR chad:ab,ti OR comoros:ab,ti OR congo:ab,ti OR ‘cote d ivoire’:ab,ti OR ‘ivory coast’:ab,ti OR congo:ab,ti OR zaire:ab,ti OR Djibouti:ab,ti OR egypt:ab,ti OR ‘equatorial guinea’:ab,ti OR ethiopia:ab,ti OR eritrea:ab,ti OR gabon:ab,ti OR gambia:ab,ti OR ghana:ab,ti OR guinea:ab,ti OR ‘guineebissau’:ab,ti OR kenya:ab,ti OR lesotho:ab,ti OR liberia:ab,ti OR libya:ab,ti OR madagascar:ab,ti OR malawi:ab,ti OR mali:ab,ti OR mauritania:ab,ti OR mauritius:ab,ti OR Mayotte:ab,ti OR morocco:ab,ti OR mozambique:ab,ti OR namibia:ab,ti OR niger:ab,ti OR nigeria:ab,ti OR reunion:ab,ti OR rwanda:ab,ti OR sahara:ab,ti OR ‘saint Helena’:ab,ti OR ‘sao tome’:ab,ti OR senegal:ab,ti OR seychelles:ab,ti OR ‘sierraleone’:ab,ti OR somalia:ab,ti OR ‘south africa’:ab,ti OR sudan:ab,ti OR swaziland:ab,ti OR togo:ab,ti OR tanzania:ab,ti OR tunisia:ab,ti OR uganda:ab,ti OR zambia:ab,ti OR zimbabwe:ab,ti OR georgia:ab,ti OR ‘solomon islands’:ab,ti OR ‘west bank’:ab,ti OR ‘gaza’:ab,ti OR kiribati:ab,ti OR ‘El Salvador’:ab,ti OR ‘caboverde’:ab,ti OR guatemala:ab,ti OR honduras:ab,ti OR nicaragua:ab,ti OR korea:ab,ti OR kyrgyz:ab,ti OR laos:ab,ti OR ‘low resource’:ab,ti OR ‘under resourced’:ab,ti OR ‘resource poor’:ab,ti OR ‘under developed’:ab,ti OR ‘underdeveloped’:ab,ti OR ‘developing country’:ab,ti OR ‘developing countries’:ab,ti OR ‘developing world’:ab,ti OR ‘third world’:ab,ti OR lmic:ab,ti OR (low:ab,ti AND middle:ab,ti AND income:ab,ti) | [1,356,](https://www.embase.com/)880 |

**Database 3: Web of Sciences**

| **Web of Sciences** | **As on Date: 31st Aug 2021** |  |
| --- | --- | --- |
|  | **Query** | **Items found** |
| #5 | #1 AND #2 AND (COVID-19 & SLUM, with LIMICs country list filter, Year 2021, 2020, 2019) | 1,190 |
| #4 | #1 AND #2 (COVID-19 & SLUM) | 2,277 |
| #1COVID-19 | (Pandemics OR “COVID-19” OR "Novel corona virus" OR coronavirus OR SARS-CoV-2 OR COVID* OR "COVID-19 Virus Infection*" OR "2019-nCoV Infection*" OR nCoV* OR "nCoV Disease" OR 2019-nCoV* OR COVID19* OR "Coronavirus Disease 2019" OR Coronavirus* OR SARS* OR "SARS-CoV-2 Infection" OR "Infection, SARS-CoV-2" OR "SARS CoV 2 Infection" OR SARS-CoV-2* OR Pandemic*) | 256,174 |
| #2SLUM | (Slum* OR underprivileged OR "Informal settlements" OR "Urban areas" OR "Urban poor" OR settlements OR "vulnerable places" OR Poverty OR "Poverty Area*" OR Ghetto* OR "Slum Area") | 284,656 |

**Database 4: ProQuest**

| **ProQuest** | **As on Date: 31st Aug 2021** |  |
| --- | --- | --- |
|  | **Query** | **Items found** |
| #5 | #1 AND #2 AND #3 (COVID-19 & SLUM & LMICs) [Filter: Scholarly journals, Humans, Male, Female) | 1,121 |
| #4 | #1 AND #2 (COVID-19 & SLUM) | 57609 |
| #1  COVID-19 | Pandemics OR COVID-19 OR "Novel corona virus" OR "Novel coronavirus" OR SARS-CoV-2 OR Coronavirus OR "COVID-19 Virus Disease*" OR "COVID-19 Virus Infection*" OR "2019-nCoV Infection*" OR nCoV* OR "Coronavirus Disease-19" OR "2019 Novel Coronavirus Disease" OR "2019 Novel Coronavirus Infection" OR "2019-nCoV Disease" OR "2019 nCoV Disease" OR 2019-nCoV* OR COVID19* OR "Coronavirus Disease 2019" OR Coronavirus* OR SARS* OR "SARS-CoV-2 Infection" OR "Infection, SARS-CoV-2" OR "SARS CoV 2 Infection" OR SARS-CoV-2* OR "COVID 19 Pandemic" | 351165 |
| #2  SLUM | Slum* OR underprivileged OR "Informal settlements" OR "Urban informal settlement" OR "Urban areas" OR "Urban poor" OR settlements OR "vulnerable places" OR "Poverty Area*" OR Poverty OR Ghetto OR Jhugg* OR Rurban OR Refugee* OR Migrant* OR Transient* OR "Disadvantaged group" | 1464972 |
| #3  LMICs | Africa OR "Central America" OR Afghanistan OR Armenia OR Bangladesh OR Bhutan OR Bolivia OR Cambodia OR Comoros OR "Georgia Republic" OR Guyana OR Haiti OR India* OR Indonesia OR Micronesia OR Kosovo OR Kyrgyzstan OR Laos OR Madagascar OR Moldova OR Mongolia OR Myanmar OR Nepal OR Pakistan OR Paraguay OR Philippines OR Samoa OR Melanesia OR "Sri Lanka" OR Syria OR Tajikistan OR Ukraine OR Uzbekistan OR Vanuatu OR Vietnam OR Yemen OR Korea OR algeria OR angola OR benin OR botswana OR "burkinafaso" OR burundi OR Cameroon OR "cape verde" OR "central african republic" OR chad OR comoros OR "cote d'ivoire" OR "ivory coast" OR congo OR zaire OR Djibouti OR egyptOR "equatorial guinea" OR ethiopia OR eritrea OR gabon OR gambia OR ghana OR guinea OR guinee-bissau OR kenya OR Lesotho OR Liberia OR Libya OR Malawi OR Mali OR Mauritania OR Mauritius OR Mayotte OR Morocco OR Mozambique OR namibia OR niger OR nigeria OR reunion OR rwanda OR sahara OR "saint Helena" OR "sao tome" OR senegal OR seychelles OR "sierraleone" OR somalia OR "south africa" OR sudan OR swaziland OR togo OR tanzania OR tunisia OR uganda OR zambia OR zimbabwe OR georgia OR "solomon islands" OR "west bank" OR gaza OR kiribati OR "El Salvador" OR "caboverde" OR guatemala OR honduras OR nicaragua OR korea OR kyrgyz OR laos OR "low resource" OR Under-resourced OR "resource poor" OR under-developed OR underdeveloped OR "Developing country" OR "Developing countries" OR "Developing world" OR "Third world" OR lmic OR "low and middle income countries" OR LMICs | 3348747 |

**Database 5: Epistemonikos**

| **Epistemonikos** | **As on Date: 31st Aug 2021** |  |
| --- | --- | --- |
|  | **Query** | **Items found** |
| #5 | #1 AND #2 AND #3 (COVID-19 & SLUM & LMICs) | 84 |
| #4 | #1 AND #2 (COVID-19 & SLUM) | **2,247** |
| #1  COVID-19 | **(**title:(pandemic) OR abstract:(pandemic)) OR (title:(epidemic) OR abstract:(epidemic)) OR (title:(COVID-19) OR abstract:(COVID-19)) OR (title:(coronavirus infection) OR abstract:(coronavirus infection)) OR (title:(COVID-19 pandemic) OR abstract:(COVID-19 pandemic)) OR (title:(2019-nCoV Infection) OR abstract:(2019-nCoV Infection)) OR (title:(2019-novel coronavirus disease) OR abstract:(2019-novel coronavirus disease)) OR (title:(2019 nCoV) OR abstract:(2019 nCoV)) OR (title:(SARS-CoV-2*) OR abstract:(SARS-CoV-2*)) | 202,751 |
| #2  SLUM | (title:(slum) OR abstract:(slum)) OR (title:(underprivileged areas) OR abstract:(underprivileged areas)) OR (title:(informal settlement*) OR abstract:(informal settlement*)) OR (title:(urban poor) OR abstract:(urban poor)) OR (title:(vulnerable population) OR abstract:(vulnerable population)) OR (title:(urban slums) OR abstract:(urban slums)) OR (title:(ghetto) OR abstract:(ghetto)) OR (title:(jhuggis) OR abstract:(jhuggis)) OR (title:(poverty areas) OR abstract:(poverty areas)) OR (title:(hovel*) OR abstract:(hovel*)) OR (title:(bustee) OR abstract:(bustee)) | 4,369 |
| #3  LMICs | Africa OR "Central America" OR "Afghanistan" OR "Armenia" OR "Bangladesh" OR "Bhutan" OR "Bolivia" OR "Cambodia" OR "Comoros" OR "Georgia (Republic)" OR "Guyana" OR "Haiti" OR "India" OR "Indonesia" OR "Micronesia" OR "Kosovo" OR "Kyrgyzstan" OR "Laos" OR "Madagascar" OR "Moldova" OR "Mongolia" OR "Myanmar" OR "Nepal" OR "Pakistan" OR "Papua the New Guinea" OR "Paraguay" OR "Philippines" OR "Samoa" OR "Melanesia" OR "Sri Lanka" OR "Syria" OR "Tajikistan" OR "East Timor" OR "Ukraine" OR "Uzbekistan" OR "Vanuatu" OR "Vietnam" OR "Yemen" OR “Korea”ORalgeria OR angola OR benin OR botswana OR burkinafaso OR burundi OR cameroon OR cape verde OR central african republic OR chad OR comoros OR “cote d'ivoire” OR ivory coast OR congo OR zaire OR Djibouti OR egypt OR equatorial guinea OR ethiopia OR eritrea OR gabon OR gambia OR ghana OR guinea OR guinee-bissau OR kenya OR lesotho OR liberia OR libya OR malawi OR mali OR mauritania OR mauritius OR Mayotte OR morocco OR mozambique OR namibia OR niger OR nigeria OR reunion OR rwanda OR sahara OR saint Helena OR sao tome OR senegal OR seychelles OR sierraleone OR somalia OR south africa OR sudan OR swaziland OR togo OR tanzania OR tunisia OR uganda OR zambia OR zimbabwe OR georgia OR "solomon islands" OR "west bank" OR "gaza" OR kiribati OR "El Salvador" OR "caboverde" OR guatemala OR honduras OR nicaragua OR korea OR "kyrgyz" OR laos OR "low resource" OR "under-resourced" OR "resource poor" OR "under-developed" OR "underdeveloped" OR "developing country" OR "developing countries" OR "developing world" OR “third world” OR lmic OR “low and middle income countries” OR LMICs | 13,232 |

**Database 6: EBSCO**

| **EBSCO** | **As on Date: 31st Aug 2021** |  |
| --- | --- | --- |
|  | **Query** | **Items found** |
| Filter: | Time: Nov 2019 to Aug 2021, Human  EBSCO (CINAHL, EBSCO Pharmacy Collection India, Academic Search Elite) | 1015 |
| #4 | #1 AND #2 (COVID-19 & SLUM) | 28,118 |
| #1  COVID-19 | Pandemics OR Influenza OR "Pandemic, 1918-1919" OR COVID-19 OR Epidemics OR "Novel cORona virus" OR Novel OR cORonavirus OR COVID-19 OR SARS-CoV-2 OR CORonavirus OR CORonavirus OR COVID-19 OR "Virus Disease*" OR COVID OR "19 Virus Disease*" OR "COVID-19 Virus Infection*" OR "2019-nCoV Infection*" OR nCoV* OR "CORonavirus Disease-19" OR S2019 "Novel CORonavirus Disease" OR S2019 "Novel CORonavirus Infection2019-nCoV Disease" OR S2019 "nCoV Disease" OR 2019-nCoV* OR COVID19* OR "CORonavirus Disease 2019" OR CORonavirus* OR SARS* OR "SARS-CoV-2 Infection" OR "Infection, SARS-CoV-2" OR "SARS CoV 2 Infection" OR SARS-CoV-2* OR "COVID 19 Pandemic" OR Pandemic* | 4,775,024 |
| #2  SLUM | (Slum) OR (underprivileged) OR (Informal settlements) OR (Urban areas) OR (Urban poor) OR (settlements) OR (vulnerable places) OR (Poverty Areas*) OR (Poverty) OR (Areas, Poverty) OR (Poverty Area) OR (Slum*) OR (Ghetto*) OR (Slum Area) | 905,490 |

**Database 7: Cochrane**

| **Cochrane** | **As on Date: 31st Aug 2021** |  |
| --- | --- | --- |
|  | **Query** | **Items found** |
| #5 | #1 AND #2 AND #3 (COVID-19 & SLUM & LMICs) | 103 |
| #4 | #1 AND #2 (COVID-19 & SLUM) | 141 |
| #1  COVID-19 | Pandemics OR “COVID-19” OR “Novel corona virus” OR “Novel coronavirus” OR “SARS-CoV-2” OR Coronavirus OR “COVID-19 Virus Disease*” OR “COVID-19 Virus Infection*” OR “2019-nCoV Infection*” OR “nCoV*” OR “Coronavirus Disease-19” OR “2019 Novel Coronavirus Disease” OR “2019 Novel Coronavirus Infection” OR “2019-nCoV Disease” OR “2019 nCoV Disease” OR 2019-nCoV* OR COVID19* OR “Coronavirus Disease 2019” OR Coronavirus* OR SARS* OR “SARS-CoV-2 Infection” OR “Infection, SARS-CoV-2” OR “SARS CoV 2 Infection” OR SARS-CoV-2* OR “COVID 19 Pandemic” | 8856 |
| #2  SLUM | Slum* OR underprivileged OR “Informal settlements” OR “Urban informal settlement” OR Urban areas OR Urban poor OR settlements OR vulnerable places OR “Poverty Area*” OR Poverty OR Ghetto OR Jhugg* OR Rurban OR Refugee* OR Migrant* OR Transient* OR “Disadvantaged group” | 25219 |
| #3  LMICs | “Africa” OR "Central America" OR "Afghanistan" OR "Armenia" OR "Bangladesh" OR "Bhutan" OR "Bolivia" OR "Cambodia" OR "Comoros" OR "Georgia Republic" OR "Guyana" OR "Haiti" OR "India* " OR "Indonesia" OR "Micronesia" OR "Kosovo" OR "Kyrgyzstan" OR "Laos" OR "Madagascar" OR "Moldova" OR "Mongolia" OR "Myanmar" OR "Nepal" OR "Pakistan" OR "Papua the New Guinea" OR "Paraguay" OR "Philippines" OR "Samoa" OR "Melanesia" OR "Sri Lanka" OR "Syria" OR "Tajikistan" OR "East Timor" OR "Ukraine" OR "Uzbekistan" OR "Vanuatu" OR "Vietnam" OR "Yemen" OR “Korea” OR algeria OR angola OR benin OR botswana OR burkinafaso OR burundi OR Cameroon OR cape verde OR central african republic OR chad OR comoros OR “cote d'ivoire” OR ivory coast OR congo OR zaire OR Djibouti OR egypt OR equatorial guinea OR ethiopia OR eritrea OR gabon OR gambia OR ghana OR guinea OR guinee-bissau OR kenya OR Lesotho OR Liberia OR Libya OR Malawi OR Mali OR Mauritania OR Mauritius OR Mayotte OR Morocco OR Mozambique OR namibia OR niger OR nigeria OR reunion OR rwanda OR sahara OR saint Helena OR sao tome OR senegal OR seychelles OR sierraleone OR somalia OR south africa OR sudan OR swaziland OR togo OR tanzania OR tunisia OR uganda OR zambia OR zimbabwe OR georgia OR "solomon islands" OR "west bank" OR "gaza" OR kiribati OR "El Salvador" OR "caboverde" OR guatemala OR honduras OR nicaragua OR korea OR "kyrgyz" OR laos OR "low resource" OR "Under-resourced" OR "resource poor" OR "under-developed" OR "underdeveloped" OR "Developing country" OR "Developing countries" OR "Developing world" OR “Third world” OR lmic OR “low and middle income countries” OR LMICs | 278486 |

**Database 8: WHO Global Index Medicus**

| **WHO Global Index Medicus** | **As on Date: 31st Aug 2021** |  |
| --- | --- | --- |
|  | **Query** | **Items found** |
| #5 | #1 AND #2 AND #3 (COVID-19 & SLUM & LMICs) | 03 |
| #4 | #1 AND #2 (COVID-19 & SLUM) | 155 |
| #1  COVID-19 | "Pandemics" OR "COVID-19" OR "Novel corona virus" OR "Novel coronavirus" OR "COVID-19" OR "SARS-CoV-2" OR "Coronavirus" OR "COVID-19" OR COVID OR "19 Virus Disease*" OR "COVID-19 Virus Infection*" OR "2019-nCoV Infection*" OR "nCoV*" OR "Coronavirus Disease-19" OR "2019 Novel Coronavirus Disease" OR "2019 Novel Coronavirus Infection" OR "2019-nCoV Disease" OR "2019 nCoV Disease" OR 2019-nCoV* OR COVID19* OR "Coronavirus Disease 2019" OR Coronavirus* OR SARS* OR "SARS-CoV-2 Infection" OR "Infection, SARS-CoV-2" OR "SARS CoV 2 Infection " OR SARS-CoV-2* OR "COVID 19 Pandemic" | 12,273 |
| #2  SLUM | (Slum) OR (underprivileged) OR (Informal settlements) OR "Urban informal settlement" OR (Urban areas) OR (Urban poor) OR (settlements) OR (vulnerable places) OR (Poverty Areas*) OR (Poverty) OR (Areas, Poverty) OR (Poverty Area) OR (Slum*) OR (Ghetto) OR (Jhugg*) OR Rurban OR (Refugee*) OR (Migrant*) OR (Transient*) OR ("Disadvantaged group") | 41,046 |
| #3  LMICs | Africa OR "Central America" OR "Afghanistan" OR "Armenia" OR "Bangladesh" OR "Bhutan" OR "Bolivia" OR "Cambodia" OR "Comoros" OR "Georgia (Republic)" OR "Guyana" OR "Haiti" OR "India* " OR "Indonesia" OR "Micronesia" OR "Kosovo" OR "Kyrgyzstan" OR "Laos" OR "Madagascar" OR "Moldova" OR "Mongolia" OR "Myanmar" OR "Nepal" OR "Pakistan" OR "Paraguay" OR "Philippines" OR "Samoa" OR "Melanesia" OR "Sri Lanka" OR "Syria" OR "Tajikistan" OR "East Timor" OR "Ukraine" OR "Uzbekistan" OR "Vanuatu" OR "Vietnam" OR "Yemen" OR "Korea" OR algeria OR angola OR benin OR botswana OR burkinafaso OR burundi OR Cameroon OR cape verde OR central african republic OR chad OR comoros OR "cote d'ivoire" OR ivory coast OR congo OR zaire OR Djibouti OR egypt OR equatorial guinea OR ethiopia OR eritrea OR gabon OR gambia OR ghana OR guinea OR guinee-bissau OR kenya OR Lesotho OR Liberia OR Libya OR Malawi OR Mali OR Mauritania OR Mauritius OR Mayotte OR Morocco OR Mozambique OR namibia OR niger OR nigeria OR reunion OR rwanda OR sahara OR saint Helena OR sao tome OR senegal OR seychelles OR sierraleone OR somalia OR south africa OR sudan OR swaziland OR togo OR tanzania OR tunisia OR uganda OR zambia OR zimbabwe OR georgia OR "solomon islands" OR "west bank" OR "gaza" OR kiribati OR "El Salvador" OR "caboverde" OR guatemala OR honduras OR nicaragua OR korea OR "kyrgyz" OR laos OR "low resource" OR "Under-resourced" OR "resource poor" OR "under-developed" OR "underdeveloped" OR "Developing country" OR "Developing countries" OR "Developing world" OR "Third world" OR lmic OR "low and middle income countries" OR LMICs | 38 |

**Database 9: 3ie Impact Evaluation Repository**

| **3ie Impact Evaluation Repository** | **As on Date: 31st Aug 2021** |  |
| --- | --- | --- |
|  | **Query** | **Items found** |
| #5 | #1 AND #2 AND #3 (COVID-19 & SLUM & LMICs) |  |
| #4 | #1 AND #2 (COVID-19 & SLUM) | 0 |
| #1COVID-19 | ("*COVID-19/pc [Prevention & Control]" OR "COVID-19" OR "coronavirus disease" OR "SARS" OR "Pandemic") | no results |
| #2SLUM | "SLUMS" OR "slum areas" OR "Slum Dwellers" OR "Slum Community" OR "Urban Slums" OR "Urban Slum" OR "Slum" OR "Slums" OR "Disadvantaged Population" OR "Vulnerable Groups" OR "Vulnerable Group" OR "Vulnerable And Underserved Populations" OR "Poor And Vulnerable Groups" OR "Migrant Population" | 157 |

**Database 10: MedRxiv and BioRxiv**

| **MedRxiv and BioRxiv** | **As on Date: 31st Aug 2021** |  |
| --- | --- | --- |
|  | **Query** | **Items found** |
| #4 | #1 AND #2 COVID-19 & SLUM"(Covid*) AND ("Slum*")" and posted between "01 Nov, 2020 and 31 Aug, 2021" | 113 |
| #1COVID-19 | "Covid*" and posted between "01 Nov, 2020 and 31 Aug, 2021" | 11,161 |
| #2SLUM | " "Slum*"" and posted between "01 Nov, 2020 and 31 Aug, 2021" | 198 |

**Appendix II**

Table 3. Quality assessment of the studies

| **Authors, Year** | **Study type** | **S1. Are there clear research questions?** | **S2. Do the collected data allow to address the research questions?** | **4.1. Is the sampling strategy relevant to address the research question?** | **4.2. Is the sample representative of the target population?** | **4.3. Are the measurements appropriate?** | **4.4. Is the risk of nonresponse bias low?** | **4.5. Is the statistical analysis appropriate to answer the research question?** |
| --- | --- | --- | --- | --- | --- | --- | --- | --- |
| Afridi et al. 2021 | Quantitative | Y | Y | Y | Y | Y | Y | Y |
| Aguilar Ticona et al. 2021 | Quantitative | Brazil | Salvador | Slum dwellers | 985 | 985 | 394 | 591 |
| Alonzo et al. 2021 | Quantitative | Y | Y | N | Y | Y | Y | Y |
| Cobre et al. 2020 | Quantitative | Y | Y | Y | Y | Y | Y | Y |
| Das et al. 2021 | Quantitative | Y | Y | N | N | Y | Y | Y |
| Islam et al. 2021 | Quantitative | Y | Y | Y | Y | Y | Y | Y |
| Jayatissa et al. 2021 | Quantitative | Y | Y | Y | N | Y | Y | Y |
| Kumar et al. 2020 | Quantitative | Y | Y | N | N | Y | Y | Y |
| Mamun and Fatima, 2021 | Quantitative | Y | Y | N | Y | Y | Y | Y |
| Muhula et al. 2021 | Quantitative | Y | Y | Y | Y | Y | Y | Y |
| Mukhopadhyay, 2020 | Quantitative | Y | Y | Y | Y | Y | Y | Y |
| Nunes et al. 2021 | Quantitative | Y | Y | Y | Y | Y | Y | Y |
| Pinchoff et al. 2021 | Quantitative | Y | Y | Y | Y | Y | Y | Y |
| Quaifeet al. 2020 | Quantitative | Y | Y | Y | Y | Y | Y | Y |
| Santana et al. 2021 | Quantitative | Y | Y | Y | Y | Y | Y | Y |
| Spiritus-Beerden et al. 2021 | Quantitative | Y | Y | Y | Y | Y | Y | Y |
|  |  | **S1. Are there clear research questions?** | **S2. Do the collected data allow to address the research questions?** | **5.1. Is there an adequate rationale for using a mixed methods design to address the research question?** | **5.2. Are the different components of the study effectively integrated to answer the research question?** | **5.3. Are the outputs of the integration of qualitative and quantitative components adequately interpreted?** | **5.4. Are divergences and inconsistencies between quantitative and qualitative results adequately addressed?** | **5.5. Do the different components of the study adhere to the quality criteria of each tradition of the methods involved?** |
| Akter et al. 2021 | Mixed method | Y | Y | Y | Y | Y | Y | Y |
| Dyalchand et al. 2021 | Mixed method | Y | Y | N | Y | Y | Y | Y |
| Guglielmi et al. 2020 | Mixed method | Y | Y | Y | Y | Y | Y | Y |
| Karp et al. 2021 | Mixed method | Y | Y | Y | Y | Y | Y | Y |
| Napier-Raman et al. 2021 | Mixed method | Y | Y | Y | Y | Y | Y | Y |
| Sumalatha et al. 2021 | Mixed method | Y | Y | Y | Y | Y | Y | Y |
|  |  | **S1. Are there clear research questions?** | **S2. Do the collected data allow to address the research questions?** | **1.1. Is the qualitative approach appropriate to answer the research question?** | **1.2. Are the qualitative data collection methods adequate to address the research question?** | **1.3. Are the findings adequately derived from the data?** | **1.4. Is the interpretation of results sufficiently substantiated by data?** | **1.5. Is there coherence between qualitative data sources, collection, analysis and interpretation?** |
| Akter et al. 2021 | Qualitative | Y | Y | Y | Y | Y | Y | Y |
| Arora and Majumder, 2021 | Qualitative | Y | Y | Y | Y | Y | Y | Y |
| Azeez et al. 2020 | Qualitative | Y | Y | Y | Y | Y | Y | Y |
| Banerjee and Rao, 2020 | Qualitative | Y | Y | Y | Y | Y | Y | Y |
| Douedari et al. 2020 | Qualitative | Y | Y | Y | Y | Y | Y | Y |
| Gichuna et al. 2020 | Qualitative | Y | Y | Y | Y | Y | Y | Y |
| Jalil et al. 2021 | Qualitative | Y | Y | Y | Y | Y | Y | Y |
| Kar et al. 2021 | Quantitative | Y | Y | Y | Y | Y | Y | Y |
| Lusambili et al. 2020 | Qualitative | Y | Y | Y | Y | Y | Y | Y |
| Mathias et al. 2020 | Qualitative | Y | Y | Y | Y | Y | Y | Y |
| Munajed and Ekren, 2020 | Qualitative | Y | Y | Y | Y | Y | Y | Y |
| Nanda, 2020 | Qualitative | Y | Y | Y | Y | Y | Y | Y |
| Oluoch-Aridi et al. 2020 | Qualitative | Y | Y | Y | Y | Y | Y | Y |
| Pandya and Redcay, 2021 | Qualitative | Y | Y | Y | Y | Y | Y | Y |
| Rashid et al. 2020 | Qualitative | Y | Y | Y | Y | Y | Y | Y |
| Zakar et al. 2021 | Qualitative | Y | Y | Y | Y | Y | Y | Y |
